# Optimization of Supercritical CO_2_ Extraction Conditions and Metabolite Composition of *Chamaepericlymenum suecicum* L. Berries from Two Locations in the Magadan Region

**DOI:** 10.3390/molecules31203658

**Published:** 2026-10-13

**Authors:** Mayya P. Razgonova, Muhammad A. Nawaz, Andrey S. Sabitov, Tamara A. Senotrusova, Natalya G. Li, Kirill S. Golokhvast

**Affiliations:** 1 N.I. Vavilov All-Russian Institute of Plant Genetic Resources, B. Morskaya 42-44, Saint-Petersburg 190000, Russia; andrsabitov@rambler.ru; 2 Advanced Engineering School, Institute of Biotechnology, Bioengineering and Food Systems, Far Eastern Federal University, Fr. Russian, pos. Ajax, 10, Vladivostok 690922, Russia; senotrusova.tale@dvfu.ru (T.A.S.); li.ng@dvfu.ru (N.G.L.); 3 Higher Engineering School of Agrobiotechnology, National Research Tomsk State University, Lenin Ave, 36, Tomsk 634050, Russia; golokhvast@sfsca.ru; 4 Laboratory for Research and Application of Supercritical Fluid Technologies in Agro-Food Biotechnology, National Research Tomsk State University, Lenin Ave, 36, Tomsk 634050, Russia; 5 Siberian Federal Scientific Centre of Agrobiotechnology RAS, 7 Centralnaya Street, Krasnoobsk 633501, Russia

**Keywords:** *Chamaepericlymenum suecicum* L., *Cornus suecica* L., Eurasian dwarf cornel, supercritical CO_2_ extraction, SFE, metabotyping, tandem mass spectrometry

## Abstract

Supercritical fluid extraction (SFE) using CO_2_ has many advantages for recovering bioactive compounds, such as high selectivity, minimal solvent use and preservation of thermolabile metabolites. This study optimized SFE for extracting *Chamaepericlymenum suecicum* (Eurasian dwarf cornel) berries and compared the metabolite profiles of samples from two locations in the Magadan region: Magadan and Ola. The extraction process was conducted at temperatures ranging from 100 to 400 °C and pressures ranging from 31 to 70 MPa, using 1% ethanol as a co-solvent. Global yield ranged from 2.60 to 8.45 mg/100 mg, with the highest yield (8.45 mg/100 mg) obtained at 250 bar and 55 °C. Two-way ANOVA revealed that temperature, pressure, and their interaction significantly influenced the extraction yield (*p* < 0.001). A total of 47 compounds were identified by HPLC-ion trap-MS/MS, including 33 polyphenolic compounds (flavones, flavonols, flavan-3-ols, anthocyanins, phenolic acids, coumarins, and lignans), as well as 14 other metabolites (including fatty acids, oxylipins, alkaloids, organic acids, sugars, naphthoquinones, and anabolic steroids). The metabolite profiles differed between the two locations: Magadan samples contained 26 unique polyphenolic compounds, while Ola samples were characterized by the presence of flavan-3-ols, which were absent in Magadan samples. Only nine compounds were shared between the two locations. The number of compounds ranged from 8 to 24, and the Shannon diversity index was higher in the Magadan samples (H′ = 3.178 and 2.303) than in the Ola samples (H′ = 2.079 and 2.398). These findings demonstrate that supercritical fluid extraction (SFE) combined with metabolomic analysis is a powerful tool for profiling *C. suecicum* berries, revealing significant geographic variation in their chemical composition and highlighting their potential as a source of bioactive compounds for functional foods and pharmaceutical applications.

## 1. Introduction

The genus *Cornus* L., which includes about 55 species, is the subject of intensive botanical study due to its unique morphology and the diversity of its ecological and geographical adaptations. Members of the genus are characterised by tetracyclic floral structures, arcuate leaf venation, and unicellular T-shaped trichomes containing calcium carbonate. Most *Cornus* species are trees or shrubs, but the subgenus Arctocrania (Endl.) Wangerin is distinguished by the presence of herbaceous flowering shoots developing from a perennial rhizome. This subgenus is characterized by chlorophyll-free scale-like leaves on the rhizome and in some nodes of the flowering stems, as well as by bristle-like structures on the outer surface of the petals [1].

Previously, the subgenus Arctocrania included two species: *Cornus canadensis* L. and *Cornus suecica* L. (*Chamaepericlymenum suecicum* L.). *C. canadensis*, described by Carl Linnaeus in 1753, has a circumboreal distribution extending from the Rocky Mountains and Appalachians in North America to the mountains of Japan. *C. suecica*, also described by Linnaeus, has a more northern and coastal circumpolar range [2]. A clear distinction between these species is difficult due to the presence of intermediate forms. Ledebour (1844) first identified the intermediate species *C. unalaschkensis*, which grows on the islands of Alaska [3]. Farr (1904) described specimens from Glacier National Park in Alaska, classifying them as *C. canadensis* var. *intermedia* and noting variability in morphological characteristics, such as leaf arrangement and coloration [4]. Hultén (1937) proposed a hypothesis about the hybrid origin of the mentioned forms, which was confirmed cytologically [5]. A detailed analysis of the morphological, chemical, phytogeographical, and cytological variability of species in the subgenus Arctocrania allowed Bayne and Denford (1979) to identify four taxa, including diploid *C. canadensis* and *C. suecica*, their hybrid *C. canadensis* × *C. suecica*, as well as the tetraploid *C. unalaschkensis*, which arose as a result of crossing *C. canadensis* and *C. suecica*. These results were supported by data on flavonoid content and cytological characteristics of the plants [5].

The flavonoid composition of *C. canadensis* and *C. suecica* demonstrates a relative simplicity of chemical structure, with a predominance of flavonol glycosides, which indicates limited chemical variability. Evolutionary changes in flavonoid content are often accompanied by a transition from woody to herbaceous forms, which is characterized by three typical changes in the flavonoid composition of leaves: the loss of proanthocyanidins, the loss of trihydroxylation in the B-ring, and the replacement of flavonols with flavones [6].

**Figure 1:**
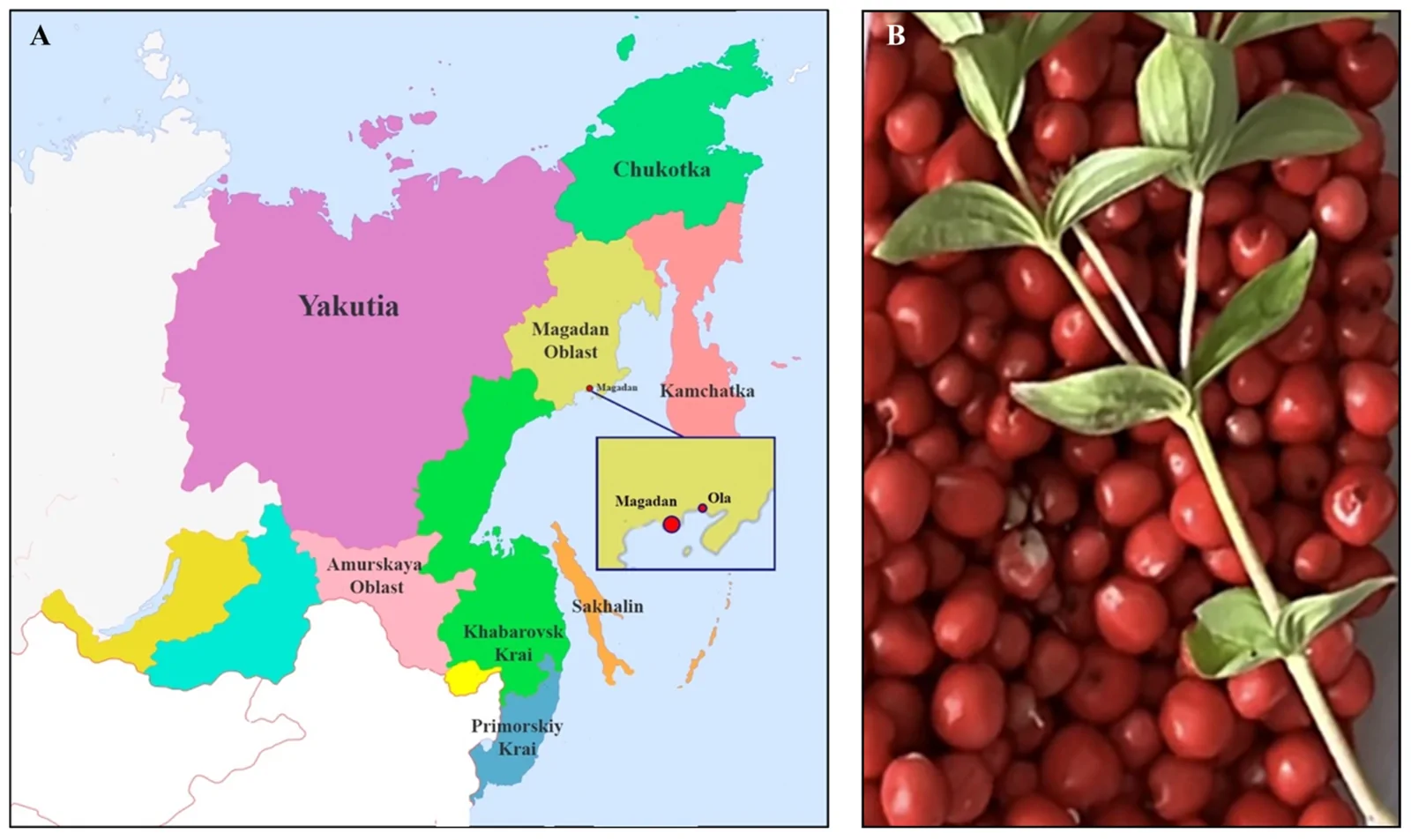
(**A**) Locations of sample collection sites of *Chamaepericlymenum suecicum* in the Magadan region. (**B**) Berries of the *Chamaepericlymenum suecicum* L. sample collected from Magadan Region. The photo was taken by A. Sabitov in August 2024 during a scientific expedition conducted in the Magadan Region.

In this work, our research group carried out an in-depth analysis of the process of extracting biologically active components from *C. suecica* (*Chamaepericlymenum suecicum* L.) berries collected in the Ecoregion of Magadan (Sea of Okhotsk) using the method of supercritical CO_2_ extraction (Figure 1). This method, which has high selectivity and the ability to effectively separate complex matrices, allowed us to minimize the loss of valuable compounds and obtain highly concentrated extracts.

National and international legislation governing the production and circulation of food products restricts the use of solvents for the extraction of food materials, allowing only a few substances of a specified degree of purity. The choice of solvent for the extraction of spices is determined by its ability to extract the desired components, the cost-effectiveness of the desolvation process, and its potential for reuse. Modern research focuses on environmentally friendly extraction technologies, such as the use of liquid CO_2_, supercritical CO_2_, pressurised liquids, and microwave extraction [7]. These methods not only reduce the harmful effects on health and the environment, but also contribute to the development of effective methods for extracting food flavors, cosmeceuticals, nutraceuticals, and biopesticides from plant raw materials. Environmentally friendly extraction technologies have the potential to meet all current regulatory requirements in the fields of safety, health, and environmental protection [8].

SFE is an advanced method based on the use of the extracting properties of carbon dioxide (CO_2_) in a supercritical state. The supercritical state of CO_2_ is characterised by unique physical and chemical properties that arise when the substance reaches its critical point. In this state, when the temperature exceeds the critical temperature (31.1 °C for CO_2_) and the pressure exceeds the critical pressure (73.8 bar for CO_2_), carbon dioxide exhibits properties intermediate between a gas and a liquid: it has high solubility comparable to liquids and low viscosity, similar to gases. These properties ensure effective dissolution of substances in the sample matrix and accelerated mass transfer between the extractant and the dissolved substance [9].

Extraction using supercritical fluids demonstrates high selectivity due to the ability to precisely control solubility by changing pressure, temperature, or a combination of both. Compared to traditional organic solvents, supercritical fluids are characterized by increased diffusivity and reduced viscosity, which optimizes the mass transfer of dissolved substances. The most commonly used supercritical fluid is carbon dioxide (CO_2_), which has unique properties: inertness, non-toxicity, non-flammability, non-explosiveness, and a high degree of purification at an economically acceptable price. These characteristics make CO_2_ an ideal solvent for extracting thermolabile components, such as carotenoids, which are susceptible to degradation under the influence of traditional organic solvents [10].

SFE is widely used in various fields, including the extraction of plant components. These technologies represent a promising direction in the development of environmentally friendly and cost-effective methods for extracting valuable components from plant raw materials [11].

Currently, information on the chemical composition and biologically active components of *C. suecicum* is limited. Due to the susceptibility of berry metabolites to degradation, SFE was employed as a gentle method of preserving labile compounds. Subsequent analysis of the obtained extracts using tandem mass spectrometry enabled the detailed identification of bioactive constituents, which provides a basis for understanding their potential biological and pharmacological significance.

## 2. Results

### 2.1. Optimization of SC-CO_2_ Extraction Yield

Supercritical CO_2_ extraction of *C. suecicum* berries was performed at different temperatures (100–400 °C) and pressures (31–70 MPa), using 1% ethanol as a co-solvent. The yield was calculated as the ratio of the dry mass of the extract to the mass of the original berry sample (mg of extract per 100 mg of berries). The global yield ranged from 2.60 to 8.45 mg per 100 mg of plant material, with an overall mean yield of 5.73 ± 1.33 mg per 100 mg.

**Figure 2:**
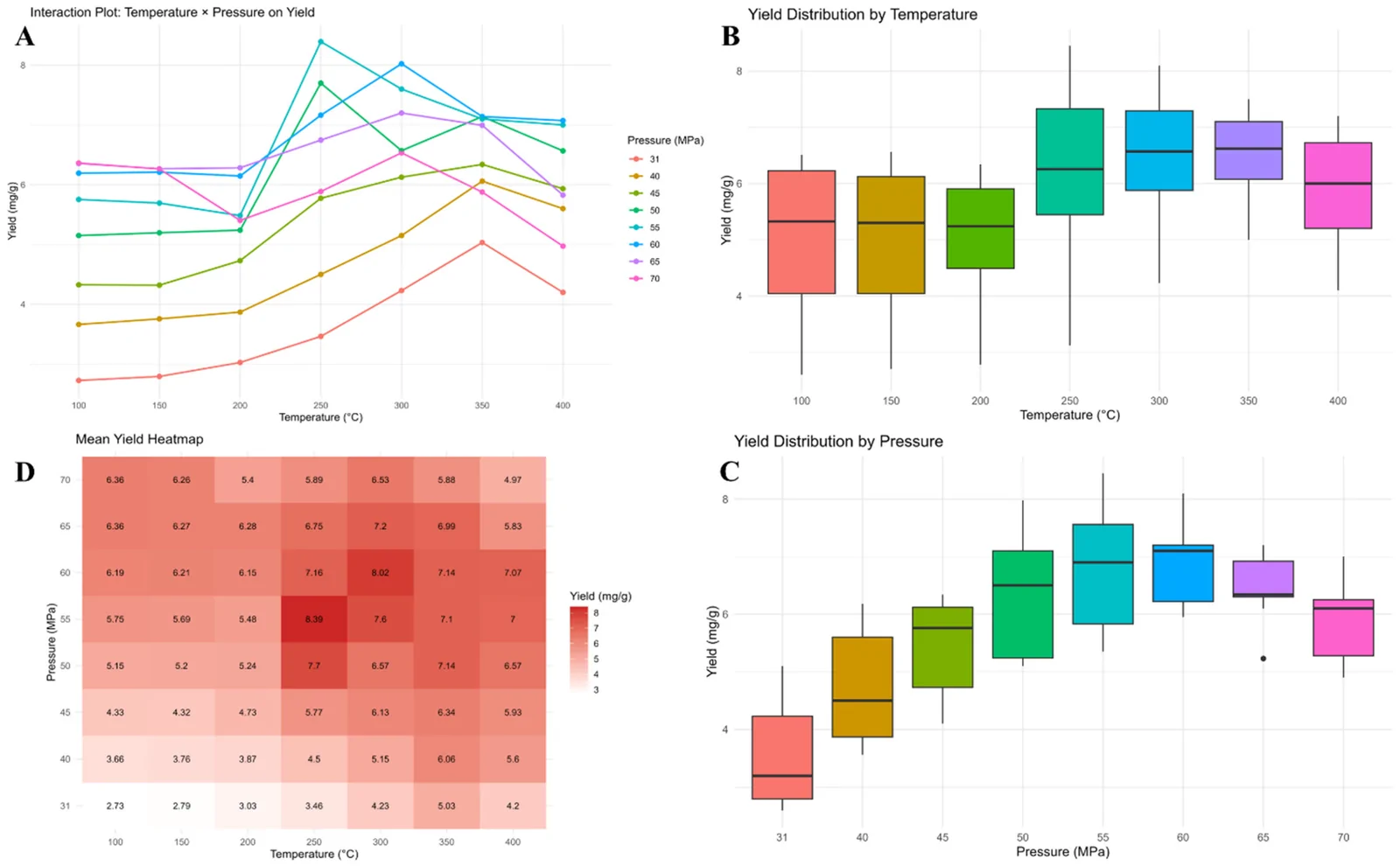
Optimization of supercritical CO_2_ extraction yield from *Chamaepericlymenum suecicum* berries. (**A**) Interaction plot showing the effect of temperature and pressure on extraction yield. Points represent mean yields; error bars indicate standard deviation. (**B**) Boxplot of extraction yield distribution by temperature. (**C**) Boxplot of extraction yield distribution by pressure. (**D**) Heatmap of mean extraction yield as a function of temperature and pressure; color gradient represents yield (mg/100 mg), with blue indicating lower yields and red indicating higher yields. Extraction conditions: pressure, 31–70 MPa; temperature, 100–400 °C; 1% ethanol as co-solvent; and extraction time, 60 min.

A two-way ANOVA revealed that temperature, pressure and their interaction had a significant influence on extraction yield (Table 1). The temperature–pressure interaction effect showed that the optimal extraction temperature depended on the applied pressure (Figure 2A). The mean yield increased progressively with temperature from 100 °C to 350 °C, reaching a maximum of 6.46 ± 0.76 mg/100 mg at 350 °C. This was followed by a slight decrease at 400 °C to 5.90 ± 0.96 mg/100 mg (Figure 2B). Similarly, yield increased with pressure from 31 to 60 MPa, peaking at 6.85 ± 0.67 mg/100 mg at 60 MPa. It then declined at higher pressures (65–70 MPa) (Figure 2C).

**Figure 3:**
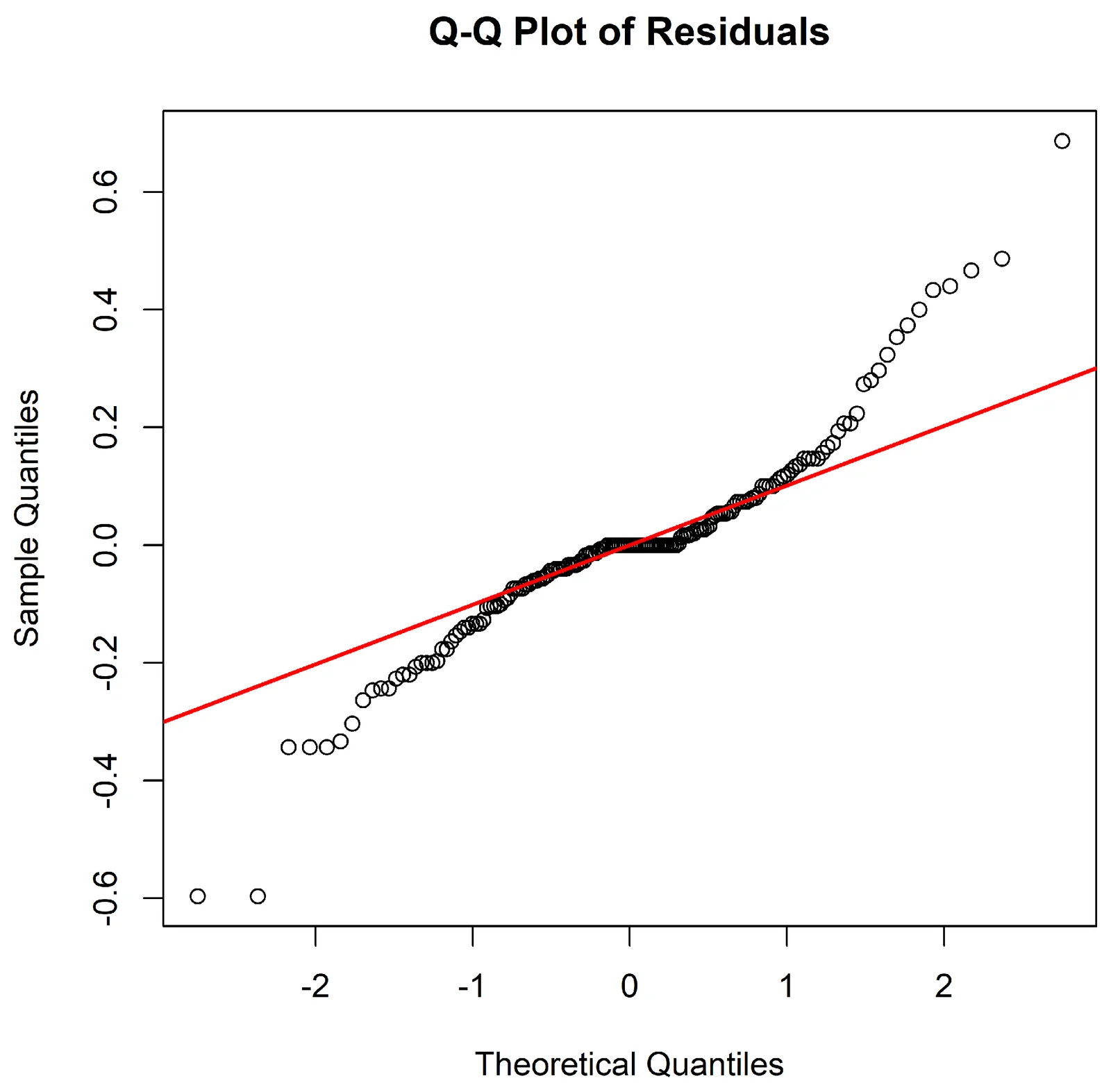
Q-Q plot of residuals from the two-way ANOVA model for SC-CO_2_ extraction yield. The red diagonal line represents the expected line under perfect normality.

The heatmap of mean yield (Figure 2D) revealed a distinct optimal region of moderate temperatures (250–300 °C) and pressures (50–60 MPa), in which yields consistently exceeded 7.5 mg/100 mg. The highest global yield was obtained at 250 °C and 55 MPa (8.39 mg/100 mg), followed by 300 °C and 60 MPa (8.02 mg/100 mg). The addition of 1% ethanol as a co-solvent enhances extraction efficiency by increasing the polarity of the supercritical fluid, thereby improving interaction with the target bioactive compounds. The QQ plot of residuals (Figure 3) confirmed that the assumptions of ANOVA were adequately met, thereby supporting the reliability of the statistical analysis.

### 2.2. Identification and Classification of Bioactive Compounds

**Figure 4:**
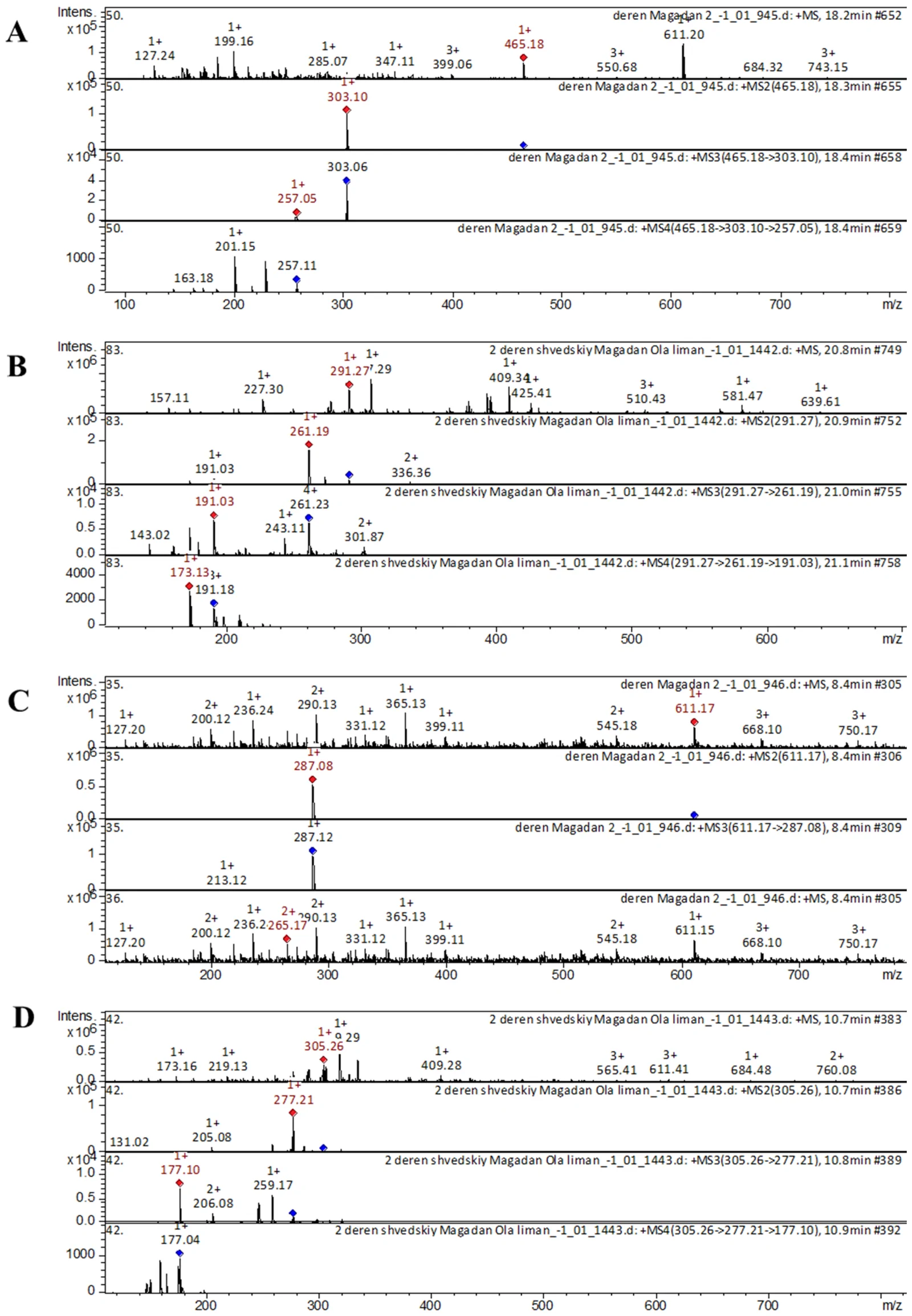
CID fragmentation spectra of representative compounds identified in supercritical CO_2_ extracts of *C. suecicum* berries: (**A**) quercetin 3-O-glucoside ([M+H]^+^ *m*/*z* 465.18); (**B**) (epi)-catechin ([M+H]^+^ *m*/*z* 291.27); (**C**) cyanidin-3-sophoroside ([M+H]^+^ *m*/*z* 611.17); (**D**) taxifolin ([M+H]^+^ *m*/*z* 305.26). For each panel, top—MS scan in the range of *m*/*z* 100–1700; bottom—fragmentation spectra (from top to bottom): MS^2^ of the protonated molecular ion, MS^3^ of the corresponding fragment ion, and MS^4^ of the subsequent fragment ion.

A total of 47 compounds were identified in SC-CO_2_ extracts of *C. suecicum* berries (Appendix A, Table A1). Thirty-three of these were polyphenolic compounds, including flavonoids, phenolic acids, coumarins and lignans. The remaining 14 compounds belonged to other chemical groups, including fatty acids, oxylipins, alkaloids, organic acids, sugars, naphthoquinones and anabolic steroids. The 33 polyphenolic compounds were classified into the following groups: flavones (three compounds: formononetin, apigenin and jaceosidin), flavonols (six compounds: quercetin, herbacetin, dihydroquercetin, myricetin, quercetin-3-glucoside and quercetin-3-hexoside), flavan-3-ols (eight compounds: catechin, epicatechin, gallocatechin and epigallocatechin, as well as their derivatives), anthocyanins (five compounds: delphinidin and cyanidin glycosides), phenolic acids (six compounds: protocatechuic acid, 3,4-dihydroxyhydrocinnamic acid, ethyl protocatechuate, methylgallic acid, ellagic acid and ellagic acid-O-hexoside), gallate esters (one compound: ethyl gallate), coumarins ((three compounds: umbelliferone, fraxetin and umbelliferone hexoside) and the lignan syringaresinol (one compound). Representative compounds from each major chemical class were confirmed by CID fragmentation spectra (Figure 4).

The mass spectra of polyphenolic compounds, including anthocyanidins, flavan-3-ols, and flavonols, which were identified in supercritical CO_2_ extracts of *C. suecicum*, are presented in Figure 4. These data are the result of a detailed analysis conducted using advanced mass spectrometry techniques, which made it possible to accurately determine the chemical composition of the samples under study. The flavonol quercetin 3-O-glucoside was found in the supercritical CO_2_ extracts of *C. suecicum* L. (Figure 4A).

The mass spectrum in positive ion mode of quercetin 3-O-glucoside from berry extracts of *C. suecicum* is shown in Figure 4A. The [M+H]^+^ ion produced one fragment ion at *m*/*z* 303.10. The fragment ion with *m*/*z* 303.10 yielded one daughter ion at *m*/*z* 257.05. The daughter ion produced two fragment ions at *m*/*z* 201.15, and *m*/*z* 163.18. This biologically active compound was successfully identified by mass-spectrometric analysis as a quercetin derivative, namely quercetin-3-O-glucoside. The identification was carried out as part of scientific research aimed at analyzing extracts from plant matrices of various plant species: *Prunus maximowiczii* [12]; *Rubus occidentalis* [13]; *Ribes meyeri* [14]; *Lonicera henryl* [15]; *Lonicera japonica* [16]; *Vaccinium myrtillus* [17]; and *Spondias purpurea* [18].

The flavan-3-ol *(epi)*-catechin was found in the extracts from berry extracts of *C. suecicum* (Figure 4B).

The mass spectrum in positive ion mode of *(epi)*-catechin from berry extracts of *C. suecicum* is shown in Figure 4B. The [M+H]^+^ ion produced two fragment ions at *m*/*z*: *m*/*z* 261.19 and *m*/*z* 191.03. The fragment ion with *m*/*z* 261.19 yielded three daughter ions: at *m*/*z* 243.11, *m*/*z* 191.03, and *m*/*z* 143.02. The chemical compound *(epi)*-catechin has been isolated and identified through mass spectrometric analysis, which demonstrates characteristic ion fragmentation patterns. This was achieved through a series of scientific studies conducted by various authors who examined the composition and properties of plant matrices: *Carpinus betulus* [19]; *Hypericum perforatum* [20]; Black soja [21]; *Glycine soja* [22]; *Rubus occidentalis* [13]; *Vaccinium myrtillus* [17]; *Aegilops trivialis* [23]; *Actinidia chinensis* [24]; *Actinidia deliciosa* [25]; Jatropha [26]; *Hibiscus rosa sinensis* [27]; and *Loropetalum chinense* [28].

The anthocyanidin cyanidin-3-sophoroside was found in the extracts from berry extracts of *C. suecicum* (Figure 4C).

The mass spectrum in positive ion mode of cyanidin-3-sophoroside from berry supercritical CO_2_ extracts of *C. suecicum* is shown in Figure 4C. The [M+H]^+^ ion produced one fragment ion at *m*/*z* 287.08. The fragment ion with *m*/*z* 287.08 yielded one daughter ion at *m*/*z* 213.12. This bioactive substance was identified in mass spectrometric studies as cyanidin-3-sophoroside in extracts of *Rubus geoides* [29]; Red currant [30]; and *Hibiscus rosa sinensis* [27].

The dihydroflavonol taxifolin was found in the extracts from supercritical CO_2_ extracts of *C. suecicum* (Figure 4D).

The mass spectrum in positive ion mode of taxifolin from supercritical CO_2_ extracts of *C. suecicum* is shown in Figure 4D. The [M+H]^+^ ion produced three fragment ions at *m*/*z* 277.21, *m*/*z* 205.08, and *m*/*z* 131.02. The fragment ion with *m*/*z* 277.21 yielded three daughter ions at *m*/*z* 259.17, *m*/*z* 206.08, and *m*/*z* 177.10. The biologically active compound taxifolin was identified through mass spectrometric analysis aimed at fragmenting the isolated chemical compounds from the following plant matrices: *Juglans mandshurica* [31], *Zea mays* [32], *Glycine soja* [22]; *Larix davurica* [33]; *Potentilla freyniana* [34]; *Millettia nitida* [35]; *Thymus vulgaris*; and Oregano [36].

### 2.3. Multivariate Analysis of Metabolite Profiles

**Figure 5:**
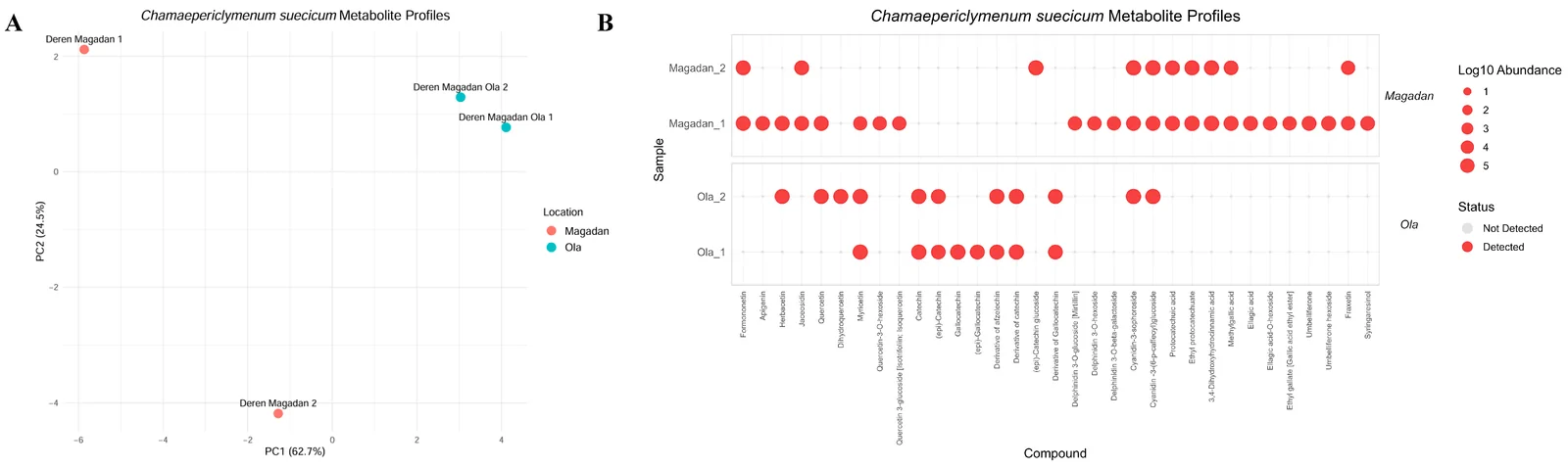
Multivariate analysis of *C. suecicum* metabolite profiles. (**A**) PCA score plot showing separation between Magadan and Ola samples based on log-transformed metabolite data. (**B**) Dot plot showing compound detection patterns and relative abundance across individual samples.

A Principal Component Analysis (PCA) was performed on the log-transformed metabolite data in order to evaluate the overall variation between the different locations. The first two principal components explained 62.7% and 24.5% of the total variance, respectively, with a cumulative percentage of 87.2% (Figure 5A). Generally, the samples from Ola showed closeness, while those from the other geography showed variation. Overall, samples from both geographies revealed a clear separation, indicating that the two geographic locations have distinct metabolic profiles.

The dot plot (Figure 5B) visualizes the patterns of compound detection and relative abundance across the individual samples. Red dots indicate detected compounds, with dot size representing log-transformed abundance, while grey dots represent non-detected compounds. The plot confirms the higher detection frequency and abundance of compounds in Magadan samples compared to Ola samples.

### 2.4. Distribution of Compounds by Geographic Location

The distribution of the 47 identified compounds (Appendix A, Table A1) varied considerably between the two collection sites, Magadan and Ola. Magadan samples exhibited greater chemical diversity, with 26 polyphenolic compounds detected exclusively in this location. These included all six phenolic acids, three coumarins, the lignan syringaresinol and several flavonoids, such as formononetin, apigenin, jaceosidin, delphinidin glycosides, quercetin 3-glucoside, quercetin 3-hexoside and ellagic acid-O-hexoside, as well as ethyl gallate.

In contrast, Ola samples were characterised by the presence of flavan-3-ols (catechin, (epi)-catechin, gallocatechin, (epi)-gallocatechin and their derivatives), which were completely absent in the Magadan samples. Additionally, dihydroquercetin and cyanidin-3-(6-p-caffeoyl)glucoside were detected in the Ola samples.

Only nine compounds were present in both locations: dihydroquercetin, cyanidin-3-(6-p-caffeoyl)glucoside, herbacetin, cyanidin-3-sophoroside, ellagic acid, quercetin-3-glucoside, quercetin, myricetin and quercetin-3-hexoside.

### 2.5. Compound Richness and Diversity

The range of compound richness (the total number of compounds detected per sample) was from eight to 24. Magadan samples exhibited higher richness (24 and 10 compounds per replicate) than Ola samples (8 and 11 compounds per replicate) (Table 2). The Shannon diversity index (H′) values ranged from 2.079 to 3.178. Magadan samples showed higher chemical diversity (H′ = 3.178 and 2.303) than Ola samples (H′ = 2.079 and 2.398).

## 3. Discussion

Significant variations in the composition of polyphenols identified in extracts of berries of *C. suecicum* collected in two geographically distant regions are attributed by the authors to differences in environmental conditions. One of the collection sites was located in close proximity to the seacoast (Magadan), while the second was significantly farther from marine areas (Ola). The scientific literature contains data indicating that marine ecosystems have a significant impact on plant metabolism, which leads to changes in the composition of bioactive compounds in extracts compared to plants growing far from the sea.

As an example, we can cite a study devoted to the polyphenolic composition of Amur grape (*Vitis amurensis*). Within the framework of this study, grape berries collected on the islands of the Sea of Japan (Peter the Great Bay) and grape berries collected on the continental territory (Primorsky Krai, Russia) were analysed. The results revealed significant differences in the composition of polyphenols between the samples, which confirms the influence of marine conditions on plant metabolism and the composition of their biologically active components [37].

The tandem mass spectrometry method, a modern analytical tool, has enabled the identification of a broad group of polyphenolic compounds in the extracts of *C. suecicum* berries. Among the identified compounds, a significant proportion are catechins, which represent an important group of flavonoids. Catechins are known for their numerous beneficial effects on human health, including antitumor, anti-adipogenic, antidiabetic, cardioprotective, antimicrobial, hepatoprotective, and neuroprotective properties. Epidemiological and clinical studies on catechins have confirmed their antitumor activity. These results have been supported by experimental data obtained in cell cultures and animal models. Moreover, the molecular mechanisms of catechins have been extensively investigated. One of the most significant mechanisms is their ability to neutralize reactive oxygen species (ROS), which play a crucial role in the pathogenesis of various diseases [38].

The biochemical properties of catechins, their antioxidant activity, and the mechanisms involved in preventing diseases associated with oxidative stress, such as cancer, cardiovascular diseases, and neurodegenerative disorders, are widely discussed in the scientific literature [39]. Catechins exhibit high antioxidant activity, making them promising candidates for developing therapeutic strategies against these diseases. In addition, catechins have anti-inflammatory properties, modulate immune response and epigenetic regulation, and inhibit the activity of receptor tyrosine kinase [40]. These properties make catechins potentially useful for the treatment of various inflammatory and autoimmune diseases.

Alzheimer’s disease (AD), one of the most common neurodegenerative diseases, is a key area of application for catechins. Oxidative stress plays an important role in the pathogenesis of AD, causing an imbalance between reactive oxygen species and antioxidants, leading to neuroinflammation and neuronal damage. Studies have shown that catechins have antioxidant and anti-inflammatory properties, as well as effects on protein kinase C activity and neurotransmission, making them promising for the prevention and treatment of AD [41].

The positive effects of catechins on neurodegenerative diseases are also supported by epidemiological evidence. Regular consumption of green tea reduces the risk of influenza infection and reduces the severity of cold symptoms. Gargling with catechin solutions may be an effective method for preventing influenza. The effects of catechins on influenza and colds require further clinical research to confirm their effectiveness. In the context of obesity, catechins such as epicatechin, methylated epigallocatechin (EGCG), theaflavins, and polyphenol metabolites found in oolong tea and black tea have been shown to have weight-reducing properties [42]. These findings highlight the potential of catechins as components of functional foods and supplements for weight management.

Significant flavonols such as herbacetin and taxifolin have been identified in the extracts of *C. suecicum* berries. Scientific studies have thoroughly examined the effects of herbacetin on myocardial damage, cardiac function, oxidative stress, and ferroptosis, as well as the corresponding signaling pathways, using a variety of experimental techniques. The results of these studies have demonstrated that herbacetin effectively reduces myocardial damage and cardiac dysfunction caused by doxorubicin, both in vivo and in vitro. At the molecular level, the action of herbacetin is manifested in the suppression of oxidative stress and lipid peroxidation through direct binding to tyrosine 342 in the long member of the acyl-CoA synthase family 4 (ACSL4). This specific interaction inhibits lipid peroxidation and ferroptosis, which allows herbacetin to be considered a promising inhibitor of ferroptosis and a potential therapeutic agent for the treatment of diseases associated with this pathological process. Thus, the obtained data highlight the significant therapeutic potential of herbacetin in the context of doxorubicin-induced cardiotoxicity, and also emphasize the importance of modulating ACSL4 activity and inhibiting lipid peroxidation in the development of strategies for treating diseases associated with ferroptosis [43].

Taxifolin (TAX), a highly active flavonoid found in a number of medicinal plants, has become the subject of intensive scientific research due to its promising potential in the treatment of cancer and cytoprotection against the toxicity induced by chemotherapy agents. TAX demonstrates unique biological properties, modulating key signaling cascades, which allows it to regulate a variety of pathophysiological processes in tumor cells.

The mechanism of action of TAX is aimed at suppressing the proliferation, migration, and invasion of tumor cells by inhibiting the cGMP-protein kinase G signaling pathway. This leads to the arrest of the cell cycle in the G1 phase, initiating apoptosis processes and thereby preventing further tumor growth. The anti-angiogenic and pro-apoptotic properties of TAX are due to the inhibition of key transcription factor expressions such as Hif1-α, as well as angiogenic factors including VEGF and AKT.

In addition, TAX demonstrates a synergistic interaction with traditional chemotherapy agents, enhancing their cytotoxic effect and reducing the resistance of tumor cells. This is achieved by suppressing the expression of P-glycoprotein, which is a key factor in multidrug resistance.

The cytoprotective properties of TAX are manifested in its ability to reduce the toxicity induced by various chemotherapy drugs. TAX effectively inhibits ferroptosis, which minimizes cisplatin-induced nephrotoxicity, cyclophosphamide- and pazopanib-induced hepatotoxicity, methotrexate-induced oral mucositis, and doxorubicin-induced cardiotoxicity. TAX’s immunomodulatory potential also deserves attention. In the tumor microenvironment, it enhances the immune response by increasing the sensitivity of tumor cells to immune checkpoint inhibitors. This opens up new prospects for combined immunotherapy of cancer diseases. In conclusion, TAX is a promising multifunctional agent with a direct antitumor effect and a powerful cytoprotective action. Its unique biological properties make it a potential candidate for widespread use in the complex therapy of cancer diseases, as well as for minimizing the side effects of chemotherapy [44].

In supercritical extracts of *C. suecicum*, the coumarins fraxetin, umbelliferone, and its hexoside have been identified. Fraxetin is a biologically active molecule that is widely distributed in various natural plants and is of significant interest in pharmacology. Fraxetin has a wide range of pharmacological properties, including antitumor, antioxidant, anti-inflammatory, hypoglycemic, anti-adipogenic, and antimicrobial effects, as well as protection of the liver, neurons, heart, bone tissue, lungs, kidneys, and other organs. Its antitumor activity may be associated with the inhibition of proliferation, invasion, and migration of malignant cells, as well as the induction of apoptosis. The beneficial effects of fraxetin on health are due to its ability to suppress cytokines and modulate the immune response, which is particularly important in the context of immune deficiency syndrome. The mechanisms of action of this molecule have been extensively studied both in vitro and in vivo, and include signaling pathways such as Janus kinase 2 (JAK2)/signal transducer and activator of transcription 3 (STAT3), phosphoinositide 3-kinase (PI3K)/protein kinase B (Akt), and nuclear factor kappa-B (NF-κB)/NLRP3. Additionally, it has been shown that fraxetin modulates the Akt/AMPK pathway, indicating its multifaceted and complex mechanism of action [45].

Umbelliferone, a coumarin compound widely distributed in higher plants, is the subject of intensive pharmacological research aimed at studying its therapeutic potential in various pathologies. Numerous studies demonstrate that this compound has multifaceted pharmacological effects, including antidiabetic, anti-cancer, anti-infective, anti-rheumatoid, and neuroprotective effects, as well as improving the condition of liver, kidney, and myocardial damage. The mechanisms of action of umbelliferone are multifaceted and include the inhibition of oxidative stress, inflammatory processes, and apoptosis. Additionally, this compound improves insulin resistance, reduces myocardial hypertrophy and tissue fibrosis, and regulates glucose levels and lipid metabolism in the blood. The most significant mechanisms of action of umbelliferone are its ability to suppress oxidative stress and inflammatory processes. These pharmacological properties make umbelliferone a promising candidate for the development of new therapeutic strategies in the treatment of a wide range of diseases [46].

As part of the scientific study, a detailed analysis was carried out on the effectiveness of the SFE method for extracting biologically active components from the berries of *C. suecicum*. The results showed that this method demonstrates a high degree of extraction of target compounds while simultaneously providing gentle conditions for the plant material. This makes it possible to preserve the native structure and biological activity of the extracted components, which is critically important for further application in the pharmaceutical and food industries.

## 4. Materials and Methods

### 4.1. Materials

The study focused on samples of *C. suecicum* berries from two locations: the vicinity of the city of Magadan, Russia (59°34′09″ north latitude, 150°48′00″ east longitude), and the vicinity of the village Ola, Magadan Region (59°34′49″ north latitude, 151°18′00″ east longitude). In August 2024, random samples of *C. suecicum* berries were collected in the designated geographical locations, with a total weight of 500 g. To ensure representativeness and increase the reliability of the experimental data, each collection was carried out twice, which made it possible to minimize possible errors and increase the statistical significance of the obtained results.

All samples were morphologically authenticated according to the current standard of Pharmacopoeia of the Eurasian Economic Union [47].

### 4.2. Chemicals and Reagents

Within the framework of this study, chemicals of analytical purity were used, which ensured high accuracy and reproducibility of the results. Acetonitrile, obtained from Fisher Scientific, located in Southborough, United Kingdom, was used as a solvent for high-performance liquid chromatography (HPLC). Formic acid, purchased from Sigma-Aldrich, located in Steinheim, Germany, was used for mass spectrometric measurements. The ultrapure water required for sample preparation was obtained using the SIEMENS ULTRA clear system, manufactured by SIEMENS Water Technologies in Munich, Germany.

### 4.3. Liquid Chromatography

High-performance liquid chromatography (HPLC) was performed using a Shimadzu LC-20 Prominence HPLC instrument (Shimadzu, Kyoto, Japan), equipped with an ultraviolet detector and a reversed-phase column with C18 silica gel (4.6 × 150 mm, particle size 2.7 mm). This method was used to separate multicomponent mixtures, ensuring high resolution and analytical accuracy. The gradient elution program, which includes two mobile phases, was carefully designed to achieve optimal separation of the analytes. Phase A used deionized water, and Phase B used acetonitrile with 0.1% formic acid by volume. The gradient program included the following stages: an initial period (0–2 min) with a phase B concentration of 0%, a linear increase in the content of phase B to 100% over 48 min (2–50 min), and a final control wash of the column with 100% phase B over 10 min (50–60 min).

The identification of compounds was carried out using a UV spectrometer SPD-20A (Shimadzu, Kyoto, Japan) at a wavelength of 220 nm, which ensured high sensitivity and selectivity of the method. The column temperature was maintained at 40 °C, and the total volumetric flow rate of the eluent was 0.25 mL/min. The volume of the sample introduced for analysis was 10 μL, which helped to minimize matrix effects and improve the accuracy of the results. For in-depth identification and confirmation of the structure of the analyzed compounds, the HPLC method was supplemented with mass spectrometry using an ion trap. This made it possible not only to determine the molecular weights of the compounds but also to obtain structural information, which significantly increased the reliability and informativeness of the study.

### 4.4. SC-CO_2_ Extraction

Supercritical carbon dioxide (CO_2_) extraction was carried out using a specialized supercritical fluid extraction device (SFE-500) manufactured by Thar SCF Waters (Milford, CT, USA). This system includes a high-pressure pump (Thar Waters P-50) designed to supply a co-solvent during the extraction of polar compounds. To accurately measure the volume of CO_2_ entering the system, a CO_2_ flow meter manufactured by Siemens (Munich, Germany) was used. Extraction vessels of varying volumes were used to extract samples of different sizes and to optimize the system’s throughput.

The CO_2_ flow rate varied within the range of 10 to 25 mL/min, while the cosolvent (C_2_H_5_OH) feed rate was 1.00 mL/min. The study object was 100 g of *C. suecicum* berries. The extraction time was recorded from the moment the critical pressure was reached under constant CO_2_ flow and amounted to 60 min. This method of supercritical fluid extraction of plant matrices was successfully tested by the authors on a variety of plant samples, including above-ground and underground parts of various plant species [37].

### 4.5. Mass Spectrometry

Mass spectrometric analysis was performed using an amaZon SL ion trap, developed by BRUKER DALTONIKS and manufactured in Munich, Germany. This instrument is equipped with an electrospray ionization source, which allows for analysis in both positive and negative ion modes. To achieve optimal analysis parameters, the following parameters were carefully adjusted: the ionization source temperature was set to 70 °C, the gas flow rate was set to 4 L/min, and the gas pressure in the nebulizer (atomizer) was set to 1.3 psi. The voltage across the capillary was 4500 V and 1500 V across the end plates, the fragmentation voltage was 280 V, and the collision energy was 60 eV.

The ion trap operated within the *m*/*z* scanning range from 100 to 1700 for MS and MS/MS methods. Chemical components were identified by comparing their retention index, mass spectra, and fragmentation results with a proprietary database created at the Laboratory of Biotechnology, Bioengineering, and Food Systems at Far Eastern Federal University, Russia. This database is based on data obtained using other spectroscopic methods, such as nuclear magnetic resonance (NMR) spectroscopy, ultraviolet spectroscopy (UV), and mass spectrometry (MS), as well as on information from literature sources, which is regularly updated and revised.

The identification of compounds was additionally confirmed by comparative analysis using such widely known databases as ChemSpider, PubChem, ChemBank, MassBank, and Elsevier ScienceDirect. Based on data regarding the exact molecular weight, characteristic fragment ions, and the results of comparisons with these databases, potential prototype components and metabolites of *C. suecicum* were successfully identified. The data acquisition rate was one spectrum per second for the MS method and two spectra per second for the MS/MS method. Data collection was carried out using specialized software developed for Windows and compatible with BRUKER DALTONIKS equipment. All experiments were repeated three times, which ensured a high degree of reproducibility of the results. To improve the accuracy of the analysis, a four-stage ion separation mode was implemented, which is standard practice in MS/MS methodology.

### 4.6. Statistical Analysis

All statistical analyses were performed using R version 4.5.3 (https://www.r-project.org/; accessed on 1 July 2026). Extraction yield data were expressed as the mean ± standard deviation (SD) from three independent replicates. The effects of temperature (100, 150, 200, 250, 300, 350 and 400 °C) and pressure (31, 40, 45, 50, 55, 60, 65 and 70 MPa), as well as the interaction between these factors, on extraction yield were evaluated using a two-way analysis of variance (ANOVA) model with replication. Prior to ANOVA, the assumptions of normality and homogeneity of variances were assessed using the Shapiro–Wilk and Levene’s tests, respectively (https://doi.org/10.5812/ijem.3505). A QQ plot of residuals was generated to visually assess normality.

For the metabolite data, the distribution was normalized by log-transforming the raw peak areas using the formula Log_10_(peak area + 1). A presence/absence matrix was then generated by converting all non-zero values to 1 while leaving zero values as they were. Compound richness was calculated as the total number of compounds detected per sample. The Shannon diversity index (H′) was computed from the presence/absence matrix using the formula H′ = −∑(pi × ln pi), where pi represents the proportion of each compound relative to the total number of compounds detected in each sample. This was done using the diversity function from the vegan package. The PCA was performed on the log-transformed metabolite data, which was mean-centered and scaled, using the prcomp function, in order to visualize clustering patterns between the two geographic locations. A dot plot was generated to visualize compound detection patterns and relative abundance across individual samples. Dot size represents log-transformed abundance, and color indicates detection status. Statistical significance was set at *p* < 0.05.

## 5. Conclusions

This study presents a detailed analysis of the SFE method and its application to metabolomic analysis of *C. suecicum* berries. SFE using CO_2_ with 1% ethanol as a co-solvent was highly efficient at extracting bioactive compounds. The optimal conditions were found to be 250 bar, 55 °C and an extraction time of 60 min, yielding 8.45 mg of extract per 100 mg of berries. The extracts contained a total of 47 compounds, including 33 polyphenolic compounds (flavones, flavonols, flavan-3-ols, anthocyanins, phenolic acids, coumarins, and lignans), as well as 14 compounds from other chemical groups (e.g., fatty acids, oxylipins, alkaloids, organic acids, sugars, naphthoquinones, and anabolic steroids). The metabolite profiles varied between the two geographic locations (Magadan and Ola), with the Magadan samples showing greater chemical diversity (26 unique compounds) than the Ola samples (11 unique compounds). Flavan-3-ols were exclusively detected in Ola samples, while phenolic acids and coumarins were unique to Magadan samples. Combining supercritical fluid extraction with metabolomic approaches is a powerful way to study the chemical composition and functional properties of *C. suecicum*. The results highlight this species’ significant potential as a raw material for developing functional food products and nutraceuticals, due to its high phenolic content, which has antioxidant, anti-inflammatory and antimicrobial properties. These findings open up new possibilities for developing innovative products in the nutraceutical and pharmaceutical industries.

## Figures and Tables

**Table 1:** Two-way ANOVA results for SC-CO_2_ extraction yield.

Source of Variation	df	Sum Sq	Mean Sq	F-Value	*p*-Value
Temperature	6	63.83	10.64	242.94	<0.001
Pressure	7	184.60	26.37	602.21	<0.001
Temperature × Pressure	42	40.00	0.95	21.75	<0.001
Residuals	112	4.90	0.04		

**Table 2:** Compound richness and Shannon diversity index for *C. suecicum* samples.

Sample	Location	Richness	Shannon Diversity (H′)
Deren Magadan (1)	Magadan	24	3.178
Deren Magadan (2)	Magadan	10	2.303
Deren Magadan Ola (1)	Ola	8	2.079
Deren Magadan Ola (2)	Ola	11	2.398

## Data Availability

The original contributions presented in this study are included in the article. Further inquiries can be directed to the corresponding authors.

## Appendix A

**Table A1:** Chemical compounds identified from the extracts of *C. suecicum* (berries) from two locations: the vicinity of the city of Magadan, Russia (59°34′09″ north latitude, 150°48′00″ east longitude), and the vicinity of the village Ola, Magadan Region (59°34′49″ north latitude, 151°18′00″ east longitude), in positive and negative ionization modes by HPLC-ion trap-MS/MS.

№	Class of Compounds	Identification	Formula	Calculated Mass	Observed Mass [M-H]^−^	Observed Mass [M+H]^+^	MS/MS Stage 1 Fragmentation	MS/MS Stage 2 Fragmentation	MS/MS Stage 3 Fragmentation	References
1	Flavone	**Formononetin** [Biochanin B; Formononetol]	C_16_H_12_O_4_	268.2641		269	256; 212; 195; 173	240; 193		*Ventilago denticulata* [48]; *Verbena officinalis* [49]; Huolisu Oral Liquid [50]; Chinese herbal formula Jian-Pi-Yi-Shen pill [51]
2	Flavone	**Apigenin** [5,7-Dixydroxy-2-(40Hydroxyphenyl)-4H-Chromen-4-One]	C_15_H_10_O_5_	270.2369		271	243; 153	197; 161		*Rosa rugosa* [52]; *Lonicera henryi* [15]; Propolis [53]; Jatropha [26]
3	Flavone	**Jaceosidin** [5,7,4′-trihydroxy-6′,3′-dimethoxyflavone]	C_17_H_14_O_7_	330.2889		331	313; 287	189; 151	119	*Artemisia argyl* [54]; *Prunus maximowiczii* [12]; *Aegilops trivialis* [23]; Mentha [55]
4	Flavonol	**Quercetin**	C_15_H_10_O_7_	302.2357		303	285; 257; 165; 121	257	229; 201; 173	*Rosa rugosa* [52]; *Capsicum annuum* [56]; *Ribes meyeri* [14]; Propolis [53]
5	Flavonol	**Herbacetin** [3,5,7,8-Tetrahydroxy-2-(4-hydro- xyphenyl)-4H-chromen-4-one]	C_15_H_10_O_7_	302.2357		303	275; 203	175		*Eleocharis dulcis* [57]; *Prunus maximowiczii* [12]; *Loropetalum chinense* [28]
6	Flavonol	**Dihydroquercetin** (Taxifolin; Taxifoliol)	C_15_H_12_O_7_	304.2516		305	277; 207	247; 207		*Glycine soja* [22]; *Larix davurica* [33]; *Juglans mandshurica* [31]
7	Flavonol	**Myricetin**	C_15_H_10_O_8_	318.2351		319	273; 219	255	209	*Taraxacum officinale* [58]; *Inula graveolens* [59]; *Juglans mandshurica* [31]; Cranberry [60]; *Prunus maximowiczii* [12]
8	Flavonol	**Quercetin 3-glucoside** [Isotrifoliin; Isoquercetin; Isoquercitrin; Hirsutrin; Quercetin-3-O-Glucopyranoside; 3-Glucosylquercetin]	C_21_H_20_O_12_	464.3763		465	303	257; 201	163	*Rubus occidentalis* [13]; *Lonicera henryi* [15]; *Lonicera japonica* [16]; *Ribes meyeri* [14]
9	Flavonol	**Quercetin 3-hexoside**	C_21_H_20_O_12_	464.3763		465	303	257	163	*Inula viscosa* [61]; *Cherimoya*; *Strawberry* [62]; *Stevia rebaudiana* [63]
10	Flavan-3-ol	**Catechin**	C_15_H_14_O_6_	290.2681		291	261; 157	191	173	*Glycine soja* [22]; *Black soja* [21]; *Ribes magellanicum* [64]; *Ribes meyeri* [14]
11	Flavan-3-ol	**(Epi)-catechin**	C_15_H_14_O_6_	290.2681		291	273; 137			*Glycine soja* [22]; *Black soja* [21]; *Vaccinium myrtillus* [17]; *Rubus occidentalis* [13]
12	Flavan-3-ol	**Gallocatechin** [+(−)Gallocatechin]	C_15_H_14_O_7_	306.2675		307	289; 261; 187; 123	243; 201; 173; 135	215; 187; 145	*G. linguiforme* [65]; *Embelia* [66]; *Carpinus betulus* [19]; *Ribes meyeri* [14]
13	Flavan-3-ol	**(Epi)-Gallocatechin**	C_15_H_14_O_7_	306.2675		307	289; 261; 187	243; 173; 135	215; 187; 145	*Carpinus betulus* [19]; *Vaccinium myrtillus* [17]; *Ribes magellanicum* [64]; *Ribes meyeri* [14]; *Loropetalum chinense* [28]
14	Flavan-3-ol	**(Epi)-afzelechin derivative**	C_18_H_16_O_10_	392.3136		393	275; 245; 215	245; 175	175; 127	PubChem
15	Flavan-3-ol	**(Epi)-catechin derivative 2**	C_18_H_16_O_11_	408.3130	407		289	243; 189	197	PubChem
16	Flavan-3-ol	**Derivative of gallocatechin**		424		425	307; 261	277; 207		PubChem
17	Flavan-3-ol	**(epi)-Catechin glucoside**	C_21_H_24_O_11_	452.4087		453	407; 341; 235	389; 347; 267; 187		*Glycine soja* [22]; *Cassia abbreviates* [67]
18	Anthocyanin	**Delphinidin 3-O-glucoside [Mirtillin]**	C_21_H_21_O_12+_	465.3905		465	303	257; 201	163	*Berberis microphylla* [68]; *Ribes magellanicum* [64]; *Prunus maximowiczii* [12]; *Loropetalum chinense* [28]
19	Anthocyanin	**Delphinidin 3-O-hexoside**	C_21_H_21_O_12+_	465.3905	463		301	255; 229; 179; 151	185; 147	*Grape vine varieties* [69]; *Myrtle* [70]
20	Anthocyanin	**Delphinidin 3-*O-beta*-galactoside**	C_21_H_21_O_12+_	465.3905		465	303	285; 257; 229; 165; 137	229; 201	*Vigna angularis* [71]; *Gaultheria antarctica* [29]; *Prunus maximowiczii* [12]
21	Anthocyanin	**Cyanidin 3-sophoroside**	C_27_H_31_O_16_	611.5335		611	287; 213	213	137	Red currant [30]; *Hibiscus rosa sinensis* [27]; *Rubus geoides* [29]
22	Anthocyanin	**Cyanidin-3-(6-p-caffeoyl)glucoside**	C_30_H_27_O_14_	611.527		611	287; 213	213	137	Gooseberry [30]
23	Hydroxybenzoic acid (Phenolic acid)	**Protocatechuic acid**	C_7_H_6_O_4_	154.1201		155	127			*Dryopteris ramosa* [72]; *Inula graveolens* [59]; *Lonicera japonica* [16]; *Ribes meyeri* [14]
24	Hydroxycinnamic acid	**3,4-Dihydroxyhydrocinnamic acid**	C_9_H_10_O_4_	182.1733		183	155	127	145	*Beta vulgaris* [73]; *Eucalyptus Globulus* [74]; *Ribes magellanicum* [64]
25	Dihydrobenzoic acid	**Ethyl protocatechuate [3,4-Dihydroxybenzoic Acid Ethyl Ester]**	C_9_H_10_O_4_	182.1733		183	155	111		*Ocimum* [75]
26	Phenolic acid	**Methylgallic acid [Methyl gallate; Methyl 3,4,5-trihydroxybenzoate]**	C_8_H_8_O_5_	184.1461		185	155; 127	127	117	*Cuphea ignea* [76]; *Ocimum* [75]; *Papaya*, *cherimoya* [62]
27	Hydroxybenzoic acid (Phenolic acid)	**Ellagic acid** [Benzoaric acid; Elagostasine; Lagistase; Eleagic acid]	C_14_H_6_O_8_	302.1926	301		257	229	201	*Cicer arietinum* [77]; *Juglans mandshurica* [31]; *Rubus occidentalis* [13]; *Ribes meyeri* [14];
28	Phenolic acid	**Ellagic acid-*O*-hexoside**	C_20_H_16_O_13_	464.3332			465	303	257; 201	*Punica granatum* [78]; *Pelargonium endlicherianum* [79]; *Carpinus betulus* [19]
29	Gallate ester	**Ethyl gallate [Gallic acid ethyl ester]**	C_9_H_10_O_5_	198.1727		199	171; 127	127		*Honey* [80]; *Oxalis corniculata* [81]; *Cicer arietinum* [77]; *Carpinus betulus* [19]
30	Coumarin	**Umbelliferone** [Skimmetin; Hydragin]	C_9_H_6_O_3_	162.1421		163	145	117		*F. glaucescens* [65]; *Phellodendron amurense* [82]; *Prunus maximowiczii* [12]
31	Coumarin	**Fraxetin**	C_10_H_8_O_5_	208.1675		209	191	117		*Actinidia chinensis* [24]; *Embelia* [66]; *Prunus maximowiczii* [12]; Jatropha [26]
32	Coumarin	**Umbelliferone hexoside**	C_15_H_16_O_8_	324.2827		325	163	145		*G. linguiforme* [65]; *Prunus maximowiczii* [12]; *Aegilops trivialis* [23]
33	Lignan	**Syringaresinol**	C_22_H_26_O_8_	418.4436		419	255	239	211	*Triticum aestivum* [83]; *Magnolia* [84]; *Prunus maximowiczii* [12]
	Others									
36	Alpha, omega dicarboxylic acid	**Sebacic acid [Decanedioic acid]**	C_10_H_18_O_4_	202.2475		203	185	139	111; 157	Jatropha [26]
37		**Tetradec-(8Z)-ene-11,13-diyn-2-one**	C_14_H_18_O	202.2476		203	185; 143	119		*Echinacea purpurea* [85]
38	Alkaloids	**N-Acetyl tryptamine** [Acetryptine]	C_12_H_14_N_2_O	202.2524		203	143			*Citrullus* spp. *Fruits* [86]; *Polygonatum odoratum* [87]
39	organic sodium salt	**Sodium ferulate**	C_10_H_9_NaO_4_	216.1658	215		179	143		*Taraxacum officinale* [58]
40	Omega-5 fatty acid	**Myristoleic acid** [Cis-9-Tetradecanoic acid]	C_14_H_26_O_2_	226.3550		227	209	192; 139	122	*F. glaucescens* [65];
41	Omega-3 fatty acid	**Stearidonic acid** [6,9,12,15-Octadecatetraenoic acid; Moroctic acid]	C_18_H_28_O_2_	276.4137		278	261; 172; 115	127		*Aegilops trivialis* [23]; *G. linguiforme*, *F. glaucescens* [65]; Jatropha [26]
42	Oxylipins	**9,10-Dihydroxy-8-oxooctadec-12-enoic acid** [oxo-DHODE; oxo-Dihydroxy-octadecenoic acid]	C_18_H_32_O_5_	328.4437	327		229; 171	211; 155	209; 167	*Brassica oleracea* var. *italica* [88]; *Bituminaria* [89]; *Taraxacum officinale* [58];
43	Oxylipin	**13-Trihydroxy-Octadecenoic acid [THODE]**	C_18_H_34_O_5_	330.4596	329		229; 211; 171; 139	211; 183; 161; 143		*Apium graveolens* [90]; *Bituminaria* [89]; Jatropha [26]
44	Alpha, omega-dicarboxylic acid	**Eicosatetraenedioic acid**	C_20_H_30_O_4_	334.4498		335	307; 289; 235	277; 207; 159	207; 159	*G. linguiforme*, *F. glaucescens* [65]; *Aegilops trivialis* [23]
45	Sugars and polyols	**Fructose 1,6-bisphosphate** [Beta-D-Fructofuranose 1,6-Bisphosphate]	C_6_H_10_O_12_P_2_	336.0839		337	273; 173	227; 173; 129		*Glycine soja* [91]; Soybean leaves [92]
46	Naphthoquinone	**8,8′-Dihydroxy-2,2′-binaphthalene-1,1′,4,4′-tetrone**	C_20_H_10_O_6_	346.2898		347	319; 247; 185; 111	291; 219; 172	261; 173	*Juglans mandshurica* [31]; *Aegilops trivialis* [23]
47	Anabolic steroid	**Vebonol**	C_30_H_44_O_3_	452.6686		453	435; 210	226; 336	210	*Rhus coriaria* [93]; *Hylosereus polyrhizus* [94]
